# The Effects of Intermittent Hypoxic Training Strategies on Maximal Oxygen Uptake in Healthy Humans: A Meta-analysis with Meta-regression

**DOI:** 10.1007/s40279-026-02482-9

**Published:** 2026-09-15

**Authors:** Dario Kohlbrenner, Abdallah Ghaith, Joey Fournier, Charles Khouri, Antonin Bordas, Samarmar Chacaroun, Monique Mendelson, Patrice Flore, Mathieu Marillier, Julien Brugniaux, Jean-Louis Pépin, Renaud Tamisier, Samuel Verges

**Affiliations:** 1 HP2 Laboratory, INSERM, Grenoble Alpes University, CHU Grenoble Alpes, Grenoble, France; 2 Epidemiology, Biostatistics, and Prevention Institute, University of Zurich, Zurich, Switzerland

## Abstract

**Background:**

Intermittent hypoxic training aims to enhance exercise performance and health, commonly assessed through maximal oxygen uptake (*V*O_2_max). Currently applied methods are live-high train-low (LHTL), live-low train-high (LLTH) and passive hypoxic conditioning (PHC). However, within these methods, numerous modes of application exist, and effectiveness is debated.

**Objective:**

To determine the effect of LHTL, LLTH and PHC on *V*O_2_max in healthy participants, and identify factors associated with the amplitude of change in *V*O_2_max.

**Methods:**

We conducted a systematic review with random-effects meta-analysis and meta-regression. Studies were identified through PubMed, EMBASE and Web of Science. We included controlled studies, calculating between-group standardised mean differences. Athlete and non-athlete populations were analysed separately. RoB2 was used to assess study quality and publication bias was investigated with funnel plots and the Egger’s test.

**Results:**

Of the 5244 identified studies, 62 were included for analysis, resulting in a sample size of 1332 participants (610 athletes, 722 non-athletes). LHTL increased *V*O_2_max significantly more than control in athletes (mean difference [MD] = 2.17; 95% CI = 0.77, 3.56) but not in non-athletes (MD = 3.1; 95% CI =  − 0.23, 6.43). LLTH did not increase *V*O_2_max significantly more than control in athletes (MD = 0.89; 95% CI =  − 0.27, 2.05) but it did in non-athletes (MD = 1.70; 95% CI = 0.85, 2.54). PHC did not increase *V*O_2_max significantly more than control in athletes (MD =  − 1.07; 95% CI =  − 3.51, 1.37) but it did in non-athletes (MD = 2.26; 95% CI = 1.00, 3.52). Multivariate meta-regressions identified the severity, duration, and frequency of hypoxic exposure to be significantly associated with the change in *V*O_2_max.

**Conclusions:**

LHTL showed a significant effect on *V*O_2_max in athletic populations while LLTH and PHC showed a significant effect on *V*O_2_max in non-athletic populations only. Future studies should target investigating which characteristics (e.g. severity, duration) of the hypoxic exposure are most beneficial.

**Supplementary Information:**

The online version contains supplementary material available at https://doi.org/10.1007/s40279-026-02482-9.

## Key Points

**Table:** 

Intermittent hypoxic training induces increases in VO2max superior to control when applied as live-high train-low (i.e. training at low altitude while spending the rest of the day in hypoxia) in athletes.
Intermittent hypoxic training induces increases in VO2max superior to control when applied as live-low train-high (i.e. perform training in hypoxia) and passive hypoxic conditioning (i.e. hypoxia at rest during wakening hours) in non-athletes only.
The severity, duration and frequency of hypoxic exposure are independently driving the observed changes in VO2max.

## Introduction

Targeting enhancement of exercise performance by using exposure to various hypoxic stimuli has been under investigation for several decades [1]. Exposed populations are heterogeneous and range from elite athletes to healthy populations. While the scope of applying hypoxia in (endurance) athlete populations focuses mainly on their already close to maximally developed aerobic capacity, thus requiring high stimuli for any further development [2], hypoxic interventions in healthy populations aim to improve their general health status. In healthy individuals, hypoxic training strategies have mainly focused on increasing maximal exercise capacity as a marker of general cardiovascular health status and as a prevention strategy for major chronic diseases (e.g. cardiovascular and metabolic disorders) [3, 4].

While severe hypoxia, both as constant and intermittent exposure, may be detrimental to human health, moderate hypoxia may elicit protective or therapeutic benefits [5–10]. Regarding beneficial effects, the physiological rationale for hypoxic interventions mainly includes improvement in oxygen transport and utilization capacities [11]. When considering hypoxic training strategies to improve fitness status, insufficient hypoxic dose (e.g. insufficient hypoxia severity or insufficient exposure duration) may lead to ineffectiveness and a waste of resources, whilst a too severe hypoxic dose may surpass the adaptation capabilities of the individual and cause various deleterious effects (e.g. fatigue, sleep and cardiovascular impairments) [12]. The severity and pattern of hypoxic exposures characterising the transition from no physiological effects to improved cardiorespiratory fitness and, ultimately, to impaired health status remain unclear and a major question regarding hypoxic training strategies for sports performance and health purposes.

In general, four methods of hypoxic exposure in combination with physical activity are described: (i) continuous hypoxic exposure during rest and exercise, also known as ‘live-high train-high’ (LHTH); (ii) hypoxic exposure during rest (mostly during sleep) while exercising in low-altitude ambient air, also known as ‘live-high train-low’ (LHTL); (iii) hypoxic exposure during exercise while spending the remaining time at low altitude, also known as ‘live-low train-high’ (LLTH); and (iv) hypoxic exposure at rest during wakening hours, also known as ‘passive hypoxic conditioning’ (PHC) [13]. Owing to LHTH being a very resource-intense and somewhat different exposure considering the continuous hypoxic stimulus applied, the present study focuses on strategies (ii) to (iv) and the effects of intermittent hypoxic strategies to improve exercise capacities.

Maximal oxygen uptake (*V*O_2_max) is known as both a major sports performance factor, especially in endurance sports [14, 15], and a highly relevant marker of health status [16, 17]. It has been suggested, for instance, that each 3.5 mL kg^−1^ min^−1^ increase in *V*O_2_max is associated with considerable (10–25%) reductions in all-cause mortality [18]. Despite the large number of studies and several meta-studies focusing on specific populations and training modalities to assess the effects of hypoxic strategies to enhance exercise capacity, the effects of intermittent hypoxic training on *V*O_2_max remain inconclusive [11, 19–22]. While some investigations showed benefits of LLTH on *V*O_2_max in sub-elite athletes only [19], a recent meta-analysis investigating repeated sprint training in hypoxia did not show a significant *V*O_2_max improvement in comparison with repeated sprint training in normoxia [23], implying that the exercise training stimulus may drive most of the adaptations. However, another meta-analysis on repeated sprint training in hypoxia found superiority regarding performance in a sport-specific intermittent running test in comparison with control [24]. Unfortunately, no analysis on *V*O_2_max was conducted in this study. Regarding LHTL, several investigations point towards a positive effect on *V*O_2_max [19, 25]. However, the authors of the meta-analysis attributed the observed changes to be likely of placebo nature [19]. An increasingly popular option to expose individuals to hypoxia is PHC. For this method, participants do not engage in any physical activity during the hypoxic exposure. All exercise training is conducted in normoxia. A network meta-analysis included PHC to evaluate its effects on *V*O_2_max, indicating PHC may be significantly more effective than a control intervention but inferior to paradigms including exercise [21]. In addition to the debate on the most effective hypoxic strategy, several key factors regarding the prescription of hypoxic training remain unknown [3]. Highly variable hypoxic doses, exposure durations, and exercise types were applied throughout literature, with inconclusive evidence on the most effective strategy.

Our aim was to comprehensively determine the effects of different types of intermittent hypoxic interventions (LHTL, LLTH and PHC) on *V*O_2_max in healthy subjects (athletes and non-athletes). In addition, we aimed to determine which factors (i.e. hypoxic dose, duration, exercise modality and intensity, etc.) were associated with the amplitude of change in *V*O_2_max for each method.

## Methods

We conducted a systematic review with meta-analysis and meta-regression in accordance with the Preferred Reporting Items for Systematic Reviews and Meta-Analysis (PRISMA) guidelines [26]. Moreover, we registered this study on PROSPERO (registration number: CRD42021222574).

### Search Strategy

We identified relevant studies published from inception until the end of October 2025 on PubMed, EMBASE and in the Web of Science Core Collection. We used combinations of the subject headings ‘hypox*’, ‘altitude’, ‘liv* high’, ‘liv* low’, ‘passive hyp* conditioning’ (see Supplementary Material), in healthy subjects, excluding studies on animal models, on patients and published in another language than English.

We included all original articles that addressed chronic (i.e. more than a single session) intermittent (i.e. < 24 h continuous exposure per day) hypoxic strategies in healthy humans, compared the intervention to an identical (active) control under normoxia (i.e. only controlled studies), reported pre- and post-measurements of *V*O_2_max (or *V*O_2_peak), assessed *V*O_2_max (or *V*O_2_peak) using conventional methods (i.e. gas exchange analyses), expressed *V*O_2_max relative to body weight and were published in English. Studies involving patients, animals, cells and continuous hypoxia exposure were excluded.

We adhered to the PRISMA guidelines for the study selection process, having all hits independently screened by two reviewers (AG and DK or AB). The two reviewers compared results and discussed discrepancies until agreement, with consultation with of third reviewer (SV) if needed. We performed two rounds of screening in this manner: (i) regarding the title and abstract, and (ii) regarding the full text.

### Data Extraction and Evidence Synthesis

We used purpose-designed data tables for data extraction. We extracted data on bibliometric details (e.g. authors, year of publication), details on study design, details on the sample studied (anthropometrics, athlete population or not) and experimental details (i.e. baseline and post-intervention *V*O_2_max, hypoxic exposure type and dose: normobaric or hypobaric, altitude or inspiratory oxygen fraction [FiO_2_], intermittent or continuous exposure, duration of exposure per day, total duration of exposure, exercise type and intensity). All data were extracted and verified by two reviewers (AG and DK or AB).

### Bias Assessment

We assessed each included study for its risk of bias according to the Cochrane Handbook for Systematic Reviews of Interventions [27]. As recommended for randomised studies, we used the Revised Tool for assessing risk of bias in randomised trials (RoB2) [28]. Two reviewers (AG and DK or AB) independently rated each included study. The two reviewers compared results and discussed discrepancies until agreement, with consultation with a third reviewer (SV) if needed. The results for RoB2 are displayed as traffic light plots for single study overview and as unweighted bar plots for a summary overview. These plots were made using the Shiny web app of the robvis visualization tool [29].

### Statistical Analysis

We performed a series of random effect meta-analyses for the three types of hypoxic exposure (i.e. LHTL, LLTH and PHC). The mean difference (MD) between normoxic and hypoxic groups was calculated for all studies. If studies consisted of several hypoxic groups, the corresponding normoxic groups were duplicated. Accounting for correlated study groups among the studies, we used a three-level hierarchical model. All outcomes were therefore analysed with a hierarchical model containing a random intercept for study groups and studies, and sub-grouped by hypoxic training strategy.

Since athlete and non-athlete populations may show distinct responses to the interventions, we performed meta-analyses according to these subgroups. The population was considered to be an athlete or non-athlete population on the basis of the study description itself and/or on the basis of *V*O_2_max. Studies with baseline values of *V*O_2_max exceeding 60 mL kg^−1^ min^−1^ were considered studying athletes. For *V*O_2_max, results are shown as mL kg^−1^ min^−1^.

We also performed multiple meta-regressions on variables of interest (i.e. age, sex, FiO_2_, duration of exposure, exercise type and intensity, blinding of participants, number of participants, athlete status and baseline *V*O_2_max). We performed univariate and adjusted meta-regressions.

Publication bias was assessed using funnel plots for mean differences in *V*O_2_max and the Egger’s regression test, as recommended in the Cochrane Handbook for Systematic Reviews of Interventions [27].

Two biostatisticians (CK and JF) performed all analyses using R 4.5.1 on MacOS (R Core Team 2025. R: a language and environment for statistical computing. R Foundation for Statistical Computing, Vienna, Austria).

## Results

### Study Characteristics

**Fig. 1:**
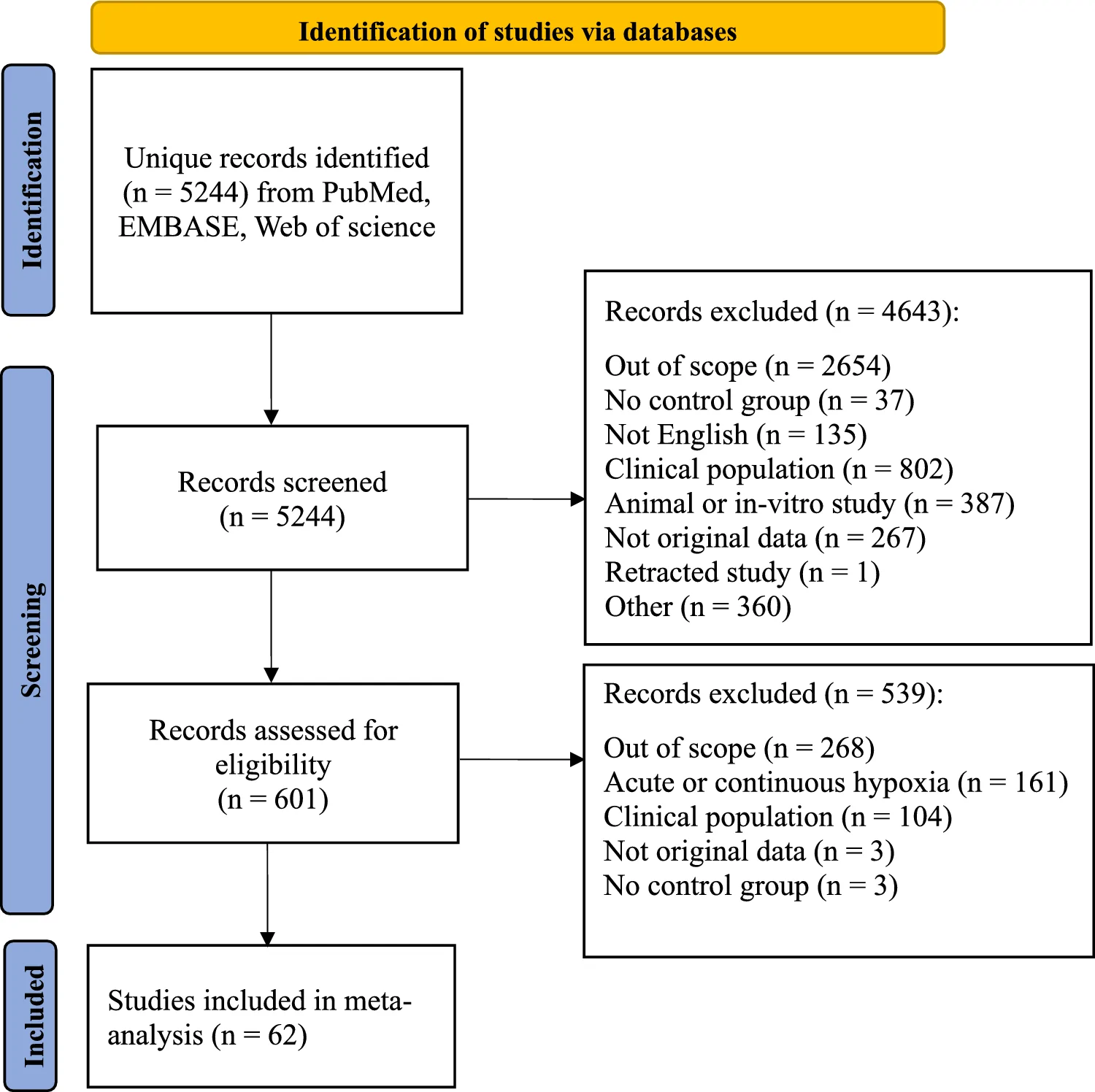
Flow diagram of the study selection process starting after de-duplication. Records screened, title and abstract screening, records assessed for eligibility and full-text screening

We identified 5244 studies and included 62 after the screening process [30–91] (Fig. 1). The meta-analysis relies on data from 1332 participants, of whom 610 were athletes and 722 were not. The characteristics of participants and interventions in each subgroup are provided in Table 1. We included 10 studies investigating LHTL [41, 44, 47, 49, 66, 74, 75, 78, 84, 89] (Table 2), 45 studies investigating LLTH [30–32, 34–40, 42, 43, 45–48, 50–53, 55, 57–65, 67, 69, 71, 72, 76, 77, 79, 82, 83, 85–88, 90, 91] (Table 3) and 11 studies investigating PHC [33, 48, 49, 54, 56, 65, 68, 70, 73, 80, 81] (Table 4). Please note that three studies employed two conditions and are thus included in two categories.

**Table 1:** Participant characteristics for each type of hypoxic intervention

	LHTL	LLTH	PHC
Number of participants (*n*)	194	884	254
Age (years)	23 (21, 24)	23 (21, 26)	23 (22, 25)
Male (%)	45	81	56
FiO_2_ (%)	17.5 (15.0, 19.0)	18.6 (14.5, 21.0)	18.0 (13.7, 21.0)
Baseline * V*O_2_max (mL kg^−1^ min^−1^)	63 (60, 67)	52 (45, 59)	42 (39, 56)
Duration per day (h)	12.5 (11.0, 14.0)	1.0 (0.5, 1.3)	1.1 (0.8, 2.0)
Total number of sessions (*n*)	19 (15, 21)	12 (9, 20)	20 (18, 20)
Total intervention duration (days)	21 (15, 28)	28 (21, 42)	28 (28, 32)
Athletes (%)	91	45	33
Proportion of blinded studies (%)	0	32	25

Data are median (Q1, Q3) or *n* (%). LHTL, live-high train-low; LLTH, live-low train-high; PHC, passive hypoxic conditioning; FiO_2_, fraction of inhaled oxygen; *V*O_2_max, maximal oxygen uptake; duration per day, number of hours in hypoxia over 24 h

**Table 2:** Characteristics of included studies with live-high train-low intervention

Study	Population	Intervention	Control	Hypoxic dose
*Studies in athlete populations*				
Park et al. [41]	Runners	Normobaric live-high train-low	Live-low train-low	21 days for > 12 h day^−1^ at 3000 m (FiO_2_ = 14.5%)
Czuba et al. [47]	Cyclists	Normobaric live-high train-low	Live-low train-low	21 days for 12 h day^−1^ at 2100 m (FiO_2_ = 16.3%)
Schmitt et al. [44]	Cross-country skiers	Normobaric live-high train-low	Live-low train-low	15 days for 14 h day^−1^ at 2700 m (FiO_2_ = 15.0%)
Schmitt et al. [44]	Cross-country skiers	Normobaric live-high train-low	Live-low train-low	15 days for 14 h day^−1^ at 2700 m (FiO_2_ = 15.0%)
Robertson et al. [66]	Runners	Normobaric live-high train-low	Live-low train-low	2 × 3 weeks for 14 h day^−1^ at 3000 m (FiO_2_ = 15.5%) (5-week washout between blocks)
Neya et al. [74]	Runners	Normobaric live-high train-low	Live-low train-low	29 days for 11 h day^−1^ at 3000 m
Robach et al. [75]	Cross-country skiers	Normobaric live-high train-low	Live-low train-low	18 days for 11 h day^−1^ at 2500, 3000 and 3500 m
Brugniaux et al. [78]	Runners	Normobaric live-high train-low	Live-low train-low	18 days for 14 h day^−1^ at 2500 m, 3000 m
Levine et al. [89]	Runners	Hypobaric live-high train-low	Live-low train-low	28 consecutive days spending all time outside training at 2500 m
Dehnert et al. [84]	Triathletes	Hypobaric live-high train-low	Live-low train-low	14 consecutive days for 13 h day^−1^ at 1956 m (FiO_2_ = 17%)
*Studies in non-athlete populations*				
Wang et al. [49]	Sedentary males	Normobaric live-high train-low	Live-low train-low	20 days for 0.5 h day^−1^ at 2733 m (FiO_2_ = 15%)

Normobaric refers to normobaric hypoxic chambers or hypoxic gas generators connected to a face mask. Hypobaric refers to hypobaric hypoxic chambers or natural altitude exposure. The hypoxic dose refers to the details provided by the studies. FiO_2_, fraction of inhaled oxygen

**Table 3:** Characteristics of studies included with live-low train-high intervention

Study	Population	Intervention	Control	Hypoxic Dose
*Studies in athletic populations*				
Zebrowska et al. [30]	Adolescent biathletes	Normobaric hypoxic training (HIIT treadmill running and HIIT arm ski ergometer)	Normoxic training (HIIT treadmill running and HIIT arm ski ergometer)	3 weeks, 3 days/week for 80 min, FiO_2_ = 15.2%
Geng et al. [32]	Pentathon athletes (men)	Normobaric hypoxic training (alternating session with long interval and HIIT treadmill running)	Normoxic training (alternating session with long interval and sprint interval treadmill running)	4 weeks, 5 days/week for 90 min, FiO_2_ = 15.3%
Giovanna et al. [36]	Endurance athletes (men)	Normobaric hypoxic training (repeated sprints on cycling ergometer)	Normoxic training (repeated sprints on cycling ergometer)	2 weeks, 3 days/week for min at 4000 m (FiO_2_ = 13%
Ambrozy et al. [38]	Boxers	Normobaric hypoxic training (boxing specific exercises)	Normoxic training (boxing specific exercises)	6 weeks, 5 days/week for 60 min at 4000 m (FiO_2_ = 12.9%)
Czuba et al. [64]	Cyclists	Normobaric hypoxic training (continuous ergometer cycling)	Normoxic training (continuous ergometer cycling)	3 weeks, 3 days/week, FiO_2_ = 15.2%
Czuba et al. [63]	Basketball players	Normobaric hypoxic training (interval treadmill running)	Normoxic training (interval treadmill running)	3 weeks, 3 days/week at 2500 m
Czuba et al. [51]	Swimmers	Normobaric hypoxic training (sprint with arm crank, HIIT ergometer cycling)	Normoxic training (sprint with arm crank, HIIT ergometer cycling)	4 weeks, 2 days/week, FiO_2_ = 15.5%
Czuba et al. [47]	Cyclists	Normobaric hypoxic training (continuous ergometer cycling)	Normoxic training (continuous ergometer cycling)	3 weeks, 3 days/week for 60–70 min at FiO_2_ = 16.3% (3000 m)
Czuba et al. [43]	Biathletes	Normobaric hypoxic training (Interval treadmill running)	Normoxic training (interval treadmill running)	3 weeks, 3 days/week FiO_2_ = 16.5%
Dufour et al. [77]	Runners	Normobaric hypoxic training (continuous treadmill running)	Normoxic training (continuous treadmill running)	6 weeks, 2 days/week, FiO_2_ = 14.5%
Kasai et al. [50]	Sprinters	Normobaric hypoxic training (repeated sprint)	Normoxic training (repeated sprint)	5 consecutive days for 1.5 h day^−1^ at 3000 m (FiO_2_ = 14.5%)
Kasai et al. [42]	Runners	Hypobaric hypoxic training (ergometer cycling)	Normoxic training (ergometer cycling)	6 consecutive days for 1.5 h day^−1^ at 3000 m (FiO_2_ = 14.5%)
Lecoultre et al. [67]	Cyclists	Normobaric hypoxic training (HIIT ergometer cycling)	Normoxic training (HIIT ergometer cycling)	4 weeks, 3 days/week at 3000 m
Millet et al. [62]	Cyclists	Normobaric hypoxic training (continuous and interval ergometer cycling)	Normoxic training (continuous and interval ergometer cycling)	3 weeks, 5 days/week for 1–1.5 h, at 3000 m
Park et al. [46]	Swimmers	Hypobaric hypoxic training (continuous treadmill running and interval ergometer cycling)	Normoxic training (continuous treadmill running and interval ergometer cycling)	6 weeks, 3 days/week for 90 min at 3000 m
Ponsot et al. [76]	Runners	Normobaric hypoxic training (continuous treadmill running)	Normoxic training (continuous treadmill running)	6 weeks, 2 days/week, FiO_2_ = 14.5%
Pramkratok et al. [35]	Rugby player	Normobaric hypoxic training (repeated sprint training)	Normoxic training (repeated sprint training)	6 weeks, 3 days/week for 45 min at FiO_2_ = 14.5%
Ramos-Campo et al. [59]	Triathletes	Normobaric hypoxic training (continuous and interval ergometer cycling)	Normoxic training (continuous and interval ergometer cycling)	7 weeks, 2 days/week for 1h, FiO_2_ = 15.0–14.5%
Roels et al. [72]	Cyclists	Normobaric hypoxic training (interval and continuous ergometer cycling)	Normoxic training (Interval and continuous ergometer cycling)	3 weeks, 5 days/week for 60–90 min, at 3000m
Roels et al. [71]	Endurance athletes	Normobaric hypoxic training (interval and continuous ergometer cycling)	Normoxic training (Interval and continuous ergometer cycling)	3 weeks, 5 days/week for 60 min, at 3000 m
Ventura et al. [82]	Cyclists	Normobaric hypoxic training (continuous ergometer cycling)	Normoxic training (continuous ergometer cycling)	6 weeks, 3 days/week for 30 min at 3200 m
*Studies in non-athlete populations*				
Allsopp et al. [37]	Undefined	Normobaric hypoxic training (resistance)	Normoxic training (resistance)	8 weeks, 2 sessions/week for 60 min at FiO2 = 14.4%
Park et al. [31]	Active men	Hypobaric hypoxic training (continuous treadmill running)	Normoxic training (continuous treadmill running)	3 weeks, 3 times/week for 60 min at 3000 m (FiO_2_ = 14.5%)
Bailey et al. [87]	Active subjects	Normobaric hypoxic training (cycling)	Normoxic training (cycling)	4 weeks, 3 times/week for 20–30 min at FiO2 = 16%
Biggs et al. [53]	Undefined	Normobaric hypoxic training (HIIT running)	Normoxic training (HIIT running)	6 weeks, 4 days/week at 2743 m
Burtscher et al. [91]	Undefined	Hypobaric hypoxic training (continuous and interval running)	Normoxic training (continuous and interval running)	12 days, daily training, 2315 m
Camacho-Cardenosa et al. [52]	Undefined	Normobaric hypoxic training (repeated sprint)	Normoxic training (repeated sprint)	4 weeks, 2 sessions/week for ~ 25 min at 3400 m (FiO_2_ = 14.6%)
Chen et al. [48]	Sedentary subjects	Normobaric hypoxic training (continuous ergometer cycling)	Normoxic training (continuous ergometer cycling)	4 weeks, 5 days/week for 30 min (FiO_2_ = 15%)
Dellavechia de Carvalho et al. [34]	Undefined	Normobaric hypoxic training (moderate intensity cycling)	Normoxic training (moderate intensity cycling)	8 weeks, 2 times/week for 50.5 min at FiO2 = 13.5%
Emonson et al. [90]	Undefined	Hypobaric hypoxic training (endurance ergometer cycling)	Normoxic training (endurance ergometer cycling)	5 weeks, 3 times/week for 45 min at ~ 2500 m
Haufe et al. [69]	Undefined	Normobaric hypoxic training (continuous treadmill running)	Normoxic training (continuous treadmill running)	4 weeks, 3 days/week for 60 min, FiO_2_ = 15%
Katayama et al. [88]	Undefined	Hypobaric hypoxic training (endurance)	Normoxic training (endurance)	2 weeks, 5 days/week for 30 min at 4500 m
Kime et al. [83]	Recreational cyclists	Normobaric hypoxic training (continuous ergometer cycling)	Normoxic training (continuous ergometer cycling)	3 weeks, 3 days/week for 2 h, FiO_2_ = 15%
Masuda et al. [86]	Sedentary subjects	Hypobaric hypoxic training (endurance)	Normoxic training (endurance)	8 weeks, 28 sessions for 1 h/session at 2500 m
Menz et al. [57]	Undefined	Normobaric hypoxic training (HIIT ergometer cycling)	Normoxic training (HIIT ergometer cycling)	3 weeks, 9 times total, FiO_2_ = 12.6%
Messonier et al. [79]	Undefined	Normobaric hypoxic training (continuous ergometer cycling)	Normoxic training (continuous ergometer cycling)	4 weeks, 6 days/week for 2 h, FiO_2_ = 13.2%
Morishima et al. [61]	Sedentary subjects	Normobaric hypoxic training (continuous cycling)	Normoxic training (continuous cycling)	4 weeks, 3 days/week for 1 h, FiO_2_ = 15%
Porcari et al. [55]	Moderately trained subjects	Normobaric hypoxic training (HIIT ergometer cycling)	Normoxic training (HIIT ergometer cycling)	6 weeks, 2 times/week for 30 min, at 914 m, 1829 m, 2743 m, 3658 m
Ramos-Campo et al. [45]	Undefined	Normobaric hypoxic training (resistance)	Normoxic training (resistance)	8 weeks, 2 days/week for 1 h (FiO_2_ = 15%)
Robach et al. [60]	Active subjects	Normobaric hypoxic training (interval ergometer cycling)	Normoxic training (interval ergometer cycling)	6 weeks, 20 sessions of 1 h in total, FiO_2_ = 15%
Vogt et al. group 1[85]	Untrained men	Normobaric hypoxic training (high-intensity ergometer cycling)	Normoxic training (high-intensity ergometer cycling)	6 weeks, 5 times/week for 30 min at 3850 m
Vogt et al. group 2 [85]	Untrained men	Normobaric hypoxic training (low-intensity ergometer cycling)	Normoxic training (low-intensity ergometer cycling)	6 weeks, 5 times/week for 30 min at 3850 m
Wang et al. [65]	Sedentary subjects	Normobaric hypoxic training (continuous ergometer cycling)	Normoxic training (continuous ergometer cycling)	4 weeks, 5 days/week for 30 min/day at ~ 2733 m (FiO_2_ = 15%)
Wang et al. [58]	Sedentary subjects	Normobaric hypoxic training (ergometer cycling)	Normoxic training (ergometer cycling)	5 weeks, 5 days/week for 30 min/day at FiO2 = 15%
Wang et al. group 1 [40]	Active subjects	Normobaric hypoxic training (ergometer cycling)	Normoxic training, blinded (interval treadmill running)	4 weeks, 2 days/week, FiO_2_ = 14.2–14.2%
Wang et al. group 2 [40]	Active subjects	Normobaric hypoxic training (ergometer cycling) and Beta-alanine supplementation	Normoxic training, (interval treadmill running) and beta-alanine supplementation	4 weeks, 2 days/week, FiO_2_ = 14.2–14.2%
Zebrowska et al. [39]	Undefined	Normobaric hypoxic training (HIIT ergometer cycling)	Normoxic training (continuous treadmill running and interval ergometer cycling)	3 weeks, 3 days/week, FiO_2_ = 15.2%

Normobaric refers to normobaric hypoxic chambers or hypoxic gas generators connected to a face mask. Hypobaric refers to hypobaric hypoxic chambers or natural altitude exposure. The hypoxic dose refers to the details provided by the studies. HIIT, high interval intensity training; FiO_2_, fraction of inhaled oxygen

**Table 4:** Characteristics of studies included with passive hypoxic conditioning intervention

Study	Population	Intervention	Control	Hypoxic dose
*Studies in athlete populations*				
Burtscher et al. [68]	Runners	Training in normoxia and intermittent normobaric hypoxia	Training in normoxia	2 × 5 weeks, 3 days/week for 2 h/day, FiO_2_ = 15–11%
Julian et al. [81]	Runners	Training in normoxia and intermittent normobaric hypoxia	Training in normoxia and normobaric normoxia	4 weeks, 5 times/week, for 70 min, 5 min hypoxia and 5 min normoxia at FiO2 = 12%, 11% and 10%
Katayama et al. [80]	Runners	Training in normoxia and normobaric hypoxia	Training in normoxia	14 days, 3 h/day FiO_2_ = 12.3%
Rodriguez et al. [73]	Swimmers and runners	Training in normoxia and hypobaric hypoxia	Training in normoxia and normobaric normoxia	4 weeks, 5 days/week, 3 h/day at 4000–5500 m
*Studies in non-athlete populations*				
Kirkaya et al. [33]	Sports science students (men)	Normobaric hypoxia	Not described	8 weeks, 3 days/week for 1 h/day at 3000 m (FiO_2_ = 14.3%)
Chen et al. [48]	Sedentary subjects	Normobaric hypoxia	Placebo altitude	5 days/week for 4 weeks for 30 min/day at 2733 m (FiO_2_ = 15%)
Nishiwaki et al. [56]	Postmenopausal women	Hypobaric hypoxia	Normobaric normoxia	8 weeks, 4 days/week for 2 h/day at 2000 m
Schega et al. [54]	Active subjects	Normobaric hypoxia followed by training on bicycle under ambient air conditions	Placebo-air mixture followed by training on bicycle under ambient air conditions	4 weeks, 3 times/week for 90 min at FiO_2_ ~ 11%
Wang et al. group 1 [70]	Sedentary subjects	Normobaric hypoxia	Placebo hypoxia	4 weeks, 5 days/week for 1 h/day FiO_2_ = 12%
Wang et al. group 2 [70]	Sedentary subjects	Normobaric hypoxia	Placebo hypoxia	4 weeks, 5 days/week for 1 h/day FiO_2_ = 15%
Wang et al. [65]	Sedentary subjects	Normobaric hypoxic	Normobatic normoxia	4 weeks, 5 days/week for 30 min/day at ~ 2733 m (FiO_2_ = 15%)
Wang et al. [49]	Sedentary subjects	Normobaric hypoxia	Normobaric normoxia	4 weeks, 5 days/week for 30 min/day at 2733 m (FiO_2_ = 15%)

Normobaric refers to normobaric hypoxic chambers or hypoxic gas generators connected to a face mask. Hypobaric refers to hypobaric hypoxic chambers or natural altitude exposure. The hypoxic dose refers to the details provided by the studies. FiO_2_, fraction of inhaled oxygen; SpO_2_, pulse oxygen saturation

### Live-High Train-Low

**Fig. 2:**
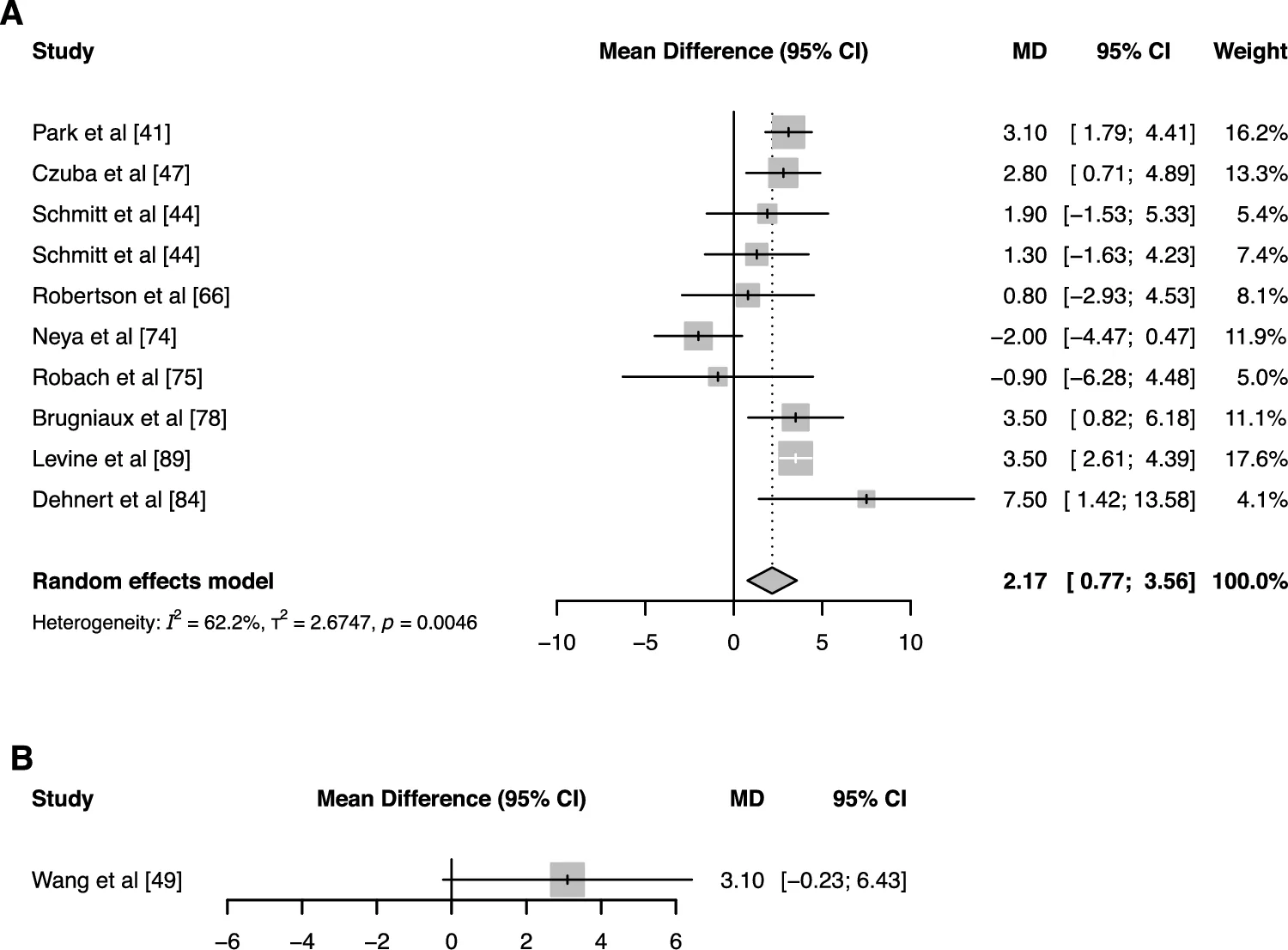
Forest plot of the mean differences in *V*O_2_max in the LHTL studies. **A** Studies in athletes. **B** Studies in non-athletes. Squares are the mean differences for the individual studies. Diamonds are the pooled mean differences across studies. *V*O_2_max, maximal oxygen uptake; MD, mean difference; 95% CI, 95% confidence interval; LHTL, live-high train-low

Regarding MD, we analysed ten between-group comparisons from nine studies concerning athletes, and one between-group comparison from one study concerning non-athletes. The meta-analysis showed significant between-group differences of LHTL on *V*O_2_max in the athlete population (MD = 2.17; 95% CI = 0.77, 3.56) and a non-significant between-group difference in the non-athlete population, in a single study only (MD = 3.10; 95% CI =  − 0.23, 6.43) (Fig. 2).

Both univariate and multivariate meta-regression for LHTL found no significant associations of the change in *V*O_2_max with the tested predictors.

### Live-Low Train-High

**Fig. 3:**
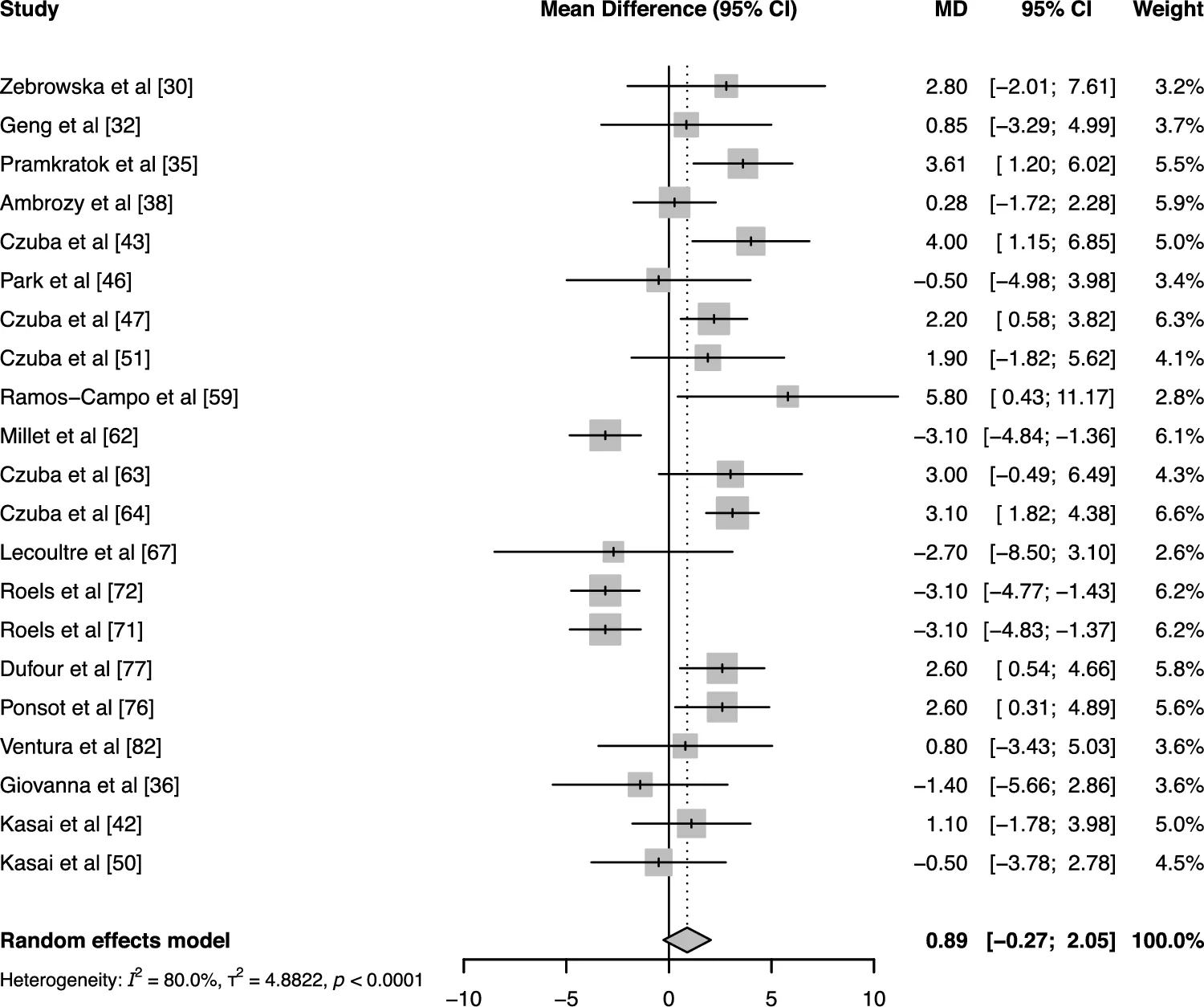
Forest plot of the mean differences in *V*O_2_max in the LLTH studies on athletes. Squares are the mean differences for the individual studies. Diamonds are the pooled mean differences across studies. *V*O_2_max, maximal oxygen uptake; MD, mean difference; 95% CI, 95% confidence interval; LLTH, live-low train-high

**Fig. 4:**
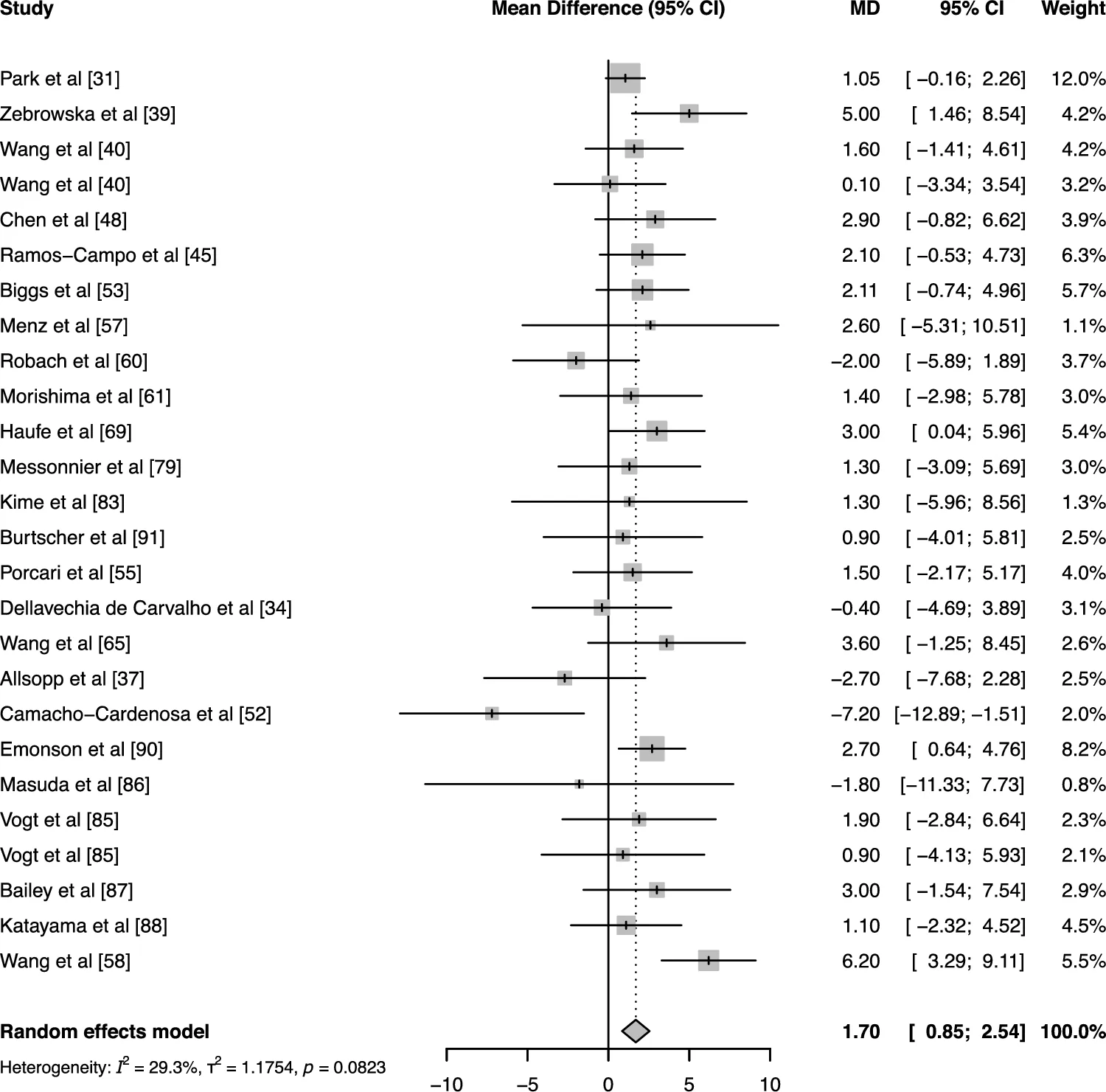
Forest plot of the mean differences in *V*O_2_max in the LLTH studies on non-athletes. Squares are the mean differences for the individual studies. Diamonds are the pooled mean differences across studies. *V*O_2_max, maximal oxygen uptake; MD, mean difference; 95% CI, 95% confidence interval; LLTH, live-low train-high

Regarding MD, we analysed 21 between-group comparisons from 21 studies concerning athletes, and 26 between-group comparisons from 24 studies concerning non-athletes. The meta-analysis showed a non-significant effect of LLTH on *V*O_2_max between-groups in the athlete population (MD = 0.89; 95% CI =  − 0.27, 2.05) and a significant effect in the non-athlete population (MD = 1.70; 95% CI = 0.85, 2.54) (Figs. 3, 4).

Univariate meta-regression for LLTH found a significant association of the change in *V*O_2_max with FiO_2_ (*B* = 0.99; 95% CI = 0.24, 1.75). The multivariate meta-regression confirmed the independent association with FiO_2_ (*B* = 0.98; 95% CI = 0.20, 1.77).

### Passive Hypoxic Conditioning

**Fig. 5:**
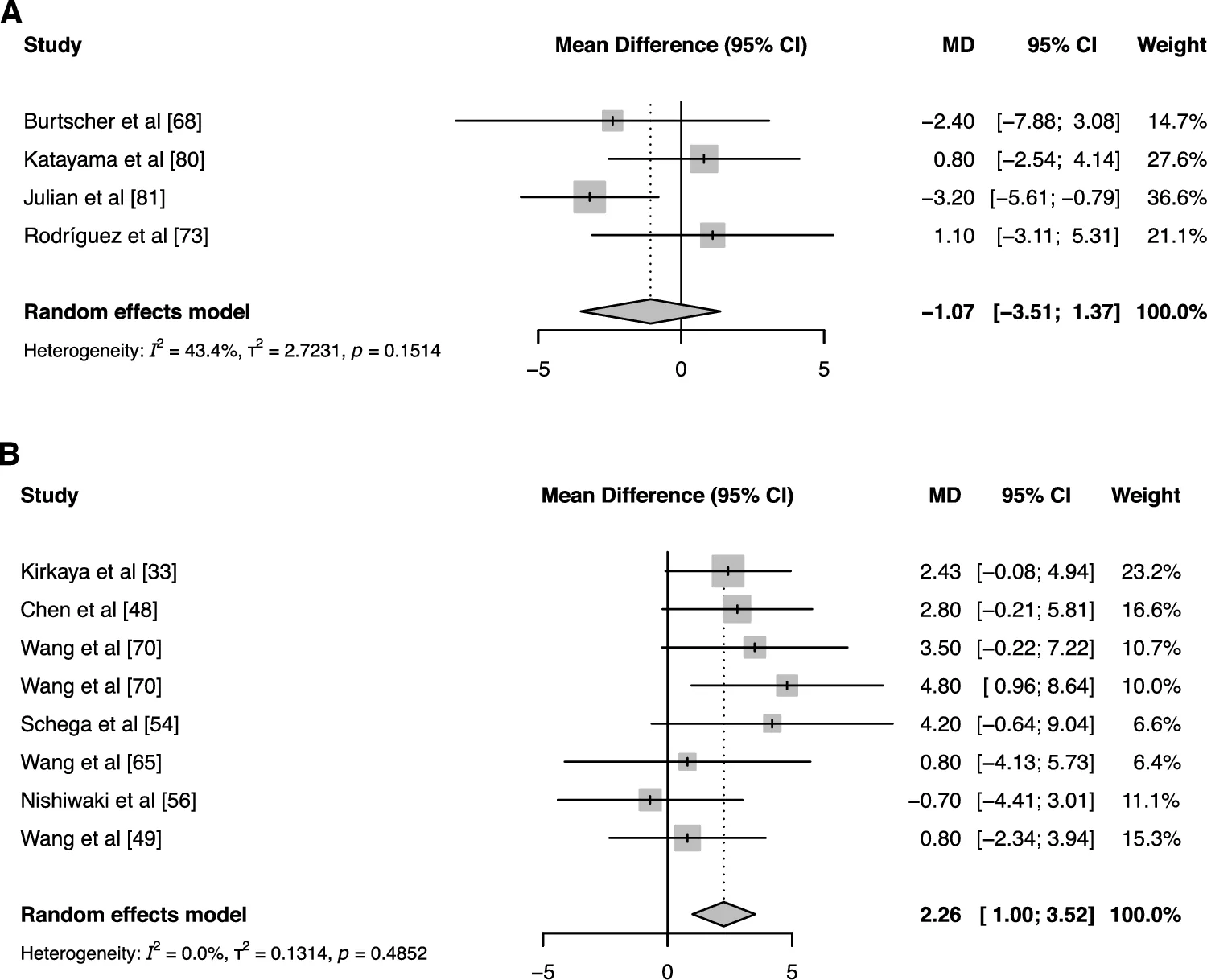
Forest plot of the mean differences in *V*O_2_max in the PHC studies. **A** Studies in athletes. **B** Studies in non-athletes. Squares are the mean differences for the individual studies. Diamonds are the pooled mean differences across studies. *V*O_2_max, maximal oxygen uptake; MD, mean difference; 95% CI, 95% confidence interval; PHC, passive hypoxic conditioning

Regarding MD, we analysed four between-group comparisons from four studies concerning athletes, and eight between-group comparisons from seven studies concerning non-athletes. The meta-analysis showed a non-significant between-group difference of PHC on *V*O_2_max in the athlete population (MD =  − 1.07; 95% CI =  − 3.51, 1.37) and a significant between-group difference in the non-athlete population (MD = 2.26; 95% CI = 1.00, 3.52) (Fig. 5).

Univariate meta-regression for PHC found significant associations of being athlete/non-athlete (*B* = 3.40; 95% CI = 0.87, 5.92), of baseline *V*O_2_max (*B* =  − 0.10; 95% CI =  − 0.19, − 0.01), of the type of exercise (*B* =  − 4.66; 95% CI =  − 7.47, − 1.85) and of sample size (*B* = 0.23; 95% CI = 0.01, 0.46) with the change in *V*O_2_max. In multivariate meta-regression, independent significant associations of hypoxic duration/day (*B* = 2.46; 95% CI = 0.07, 4.85) and the frequency of hypoxic exposure (*B* =  − 0.28; 95% CI =  − 0.54, − 0.02) with the change in *V*O_2_max were found.

### Risk of Bias

**Fig. 6:**
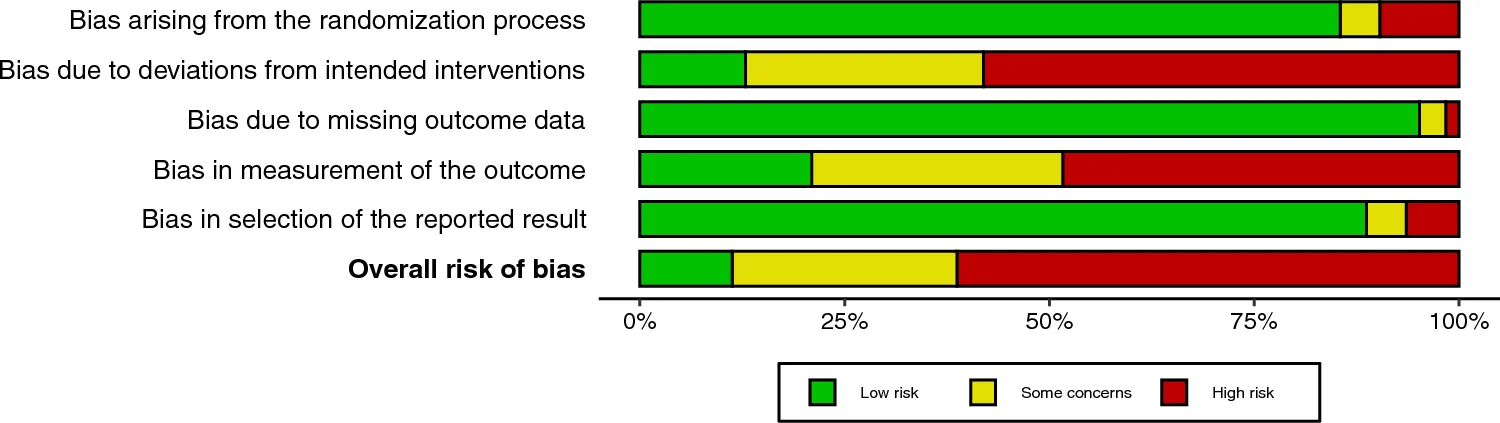
Bar plot for the summary overview of the revised tool for assessing risk of bias in randomised trials (RoB2)

The RoB2 showed some concerns or high risk for overall risk of bias in a majority of the studies included in this work (Fig. 6). A majority of studies experienced low risk of bias from the randomisation process, from missing outcome data and from selection of the reported result. An elevated risk of bias was present owing to possible deviations from the intended interventions and measurement of the outcome (Supplementary Fig. S1).

The funnel plot for mean differences in *V*O_2_max is presented in Supplementary Fig. S2. Visual inspection determines no relevant asymmetry, which is confirmed by the non-significant Egger test (*p* = 0.67).

## Discussion

We report on the effects of intermittent hypoxic training strategies on *V*O_2_max in athlete and non-athlete populations. For athletes, LHTL induces significantly larger changes in *V*O_2_max than control while LLTH and PHC do not induce changes in *V*O_2_max significantly different to control. For non-athletes, LLTH and PHC induce significantly larger changes in *V*O_2_max than control while LHTL does not. In addition, our meta-regressions identified FiO_2_ (in LLTH), hypoxic duration/day and frequency of hypoxic exposure as independent predictors for changes in *V*O_2_max.

Regarding the LHTL approach, significant between-group differences were discovered in athlete populations only. We identified only a single study fulfilling inclusion criteria investigating non-athletes, therefore preventing clear conclusions being drawn in this population. Logistical difficulties and inconveniences of LHTL are probably the reason for the lack of studies in non-athletes, since LHTL usually requires participants to spend their nights away from home, in either hypoxic chambers or tents, or to ascend to altitude in the evening and descend the following morning. One mechanism that might contribute to the increased *V*O_2_max following LHTL in athletes is an improvement in oxygen transport capacity as illustrated by an increase in haemoglobin mass [92], although this is not a universal finding [93]. Despite the analysed LHTL studies applying various durations and severities of hypoxic exposure (FiO_2_ ranging from 14.5 to 17%, daily hypoxic exposure ranging from 11 to 14 h and total duration ranging from 15 to 29 days), the meta-regression could not identify LHTL characteristics associated with changes in *V*O_2_max. It should also be underlined that LHTL studies are prone to placebo effects [94] and lack of blinding contributes substantially to the overall high risk of bias in the included studies. Further studies, applying a double-blind design, are therefore required to clarify the optimal characteristics of LHTL to enhance aerobic capacities and athletic performances.

Regarding LLTH, our meta-analysis suggests a significant effect in non-athletes only. Despite the amount of additional gain in *V*O_2_max from LLTH being relatively small (MD = 1.70 mL kg^−1^ min^−1^) from a performance perspective, it may be interesting in certain populations with low baseline values or also be applied in rehabilitation settings. In addition, our meta-regression showed FiO_2_ to be significantly associated with the change in *V*O_2_max. Accordingly, future studies on the topic should consider applying hypoxic stimuli at the more severe end of the spectrum. Moreover, the effect of exercise training performed under hypoxia may be influenced by the exercise type and intensity, e.g. continuous, interval or sprint exercise training [20, 21, 23]. Most studies included in our analysis applied continuous or interval training, and only a few applied sprints or very short intervals. However, when considering the type of exercise performed in hypoxia (i.e. continuous versus interval/sprint exercise), we could not detect differences in *V*O_2_max improvements associated with the exercise training modality. As a physiological basis, hypoxic exercise training may trigger specific muscle adaptations including increased capillary density, myoglobin content, oxidative enzyme activity, buffering capacities and muscle efficiency that might potentiate the increase in *V*O_2_max associated with normoxic exercise training [12, 95, 96]. The lack of significant effect of LLTH on *V*O_2_max in athletes may suggest that these muscle adaptations may already be highly developed in this population due to high training load for numerous years. However, this lack of significant increase in *V*O_2_max does not rule out an improvement in sport-specific performance following LLTH in athletes [24]. Finally, also with LLTH, the possibility of placebo effects needs to be acknowledged. A recent study attempted to quantify the placebo effects in LLTH but failed in the absence of any hypoxia-related differences between the groups [97].

Regarding PHC, non-athletes seem to receive a relatively large benefit from the hypoxic exposure (MD = 2.26 mL kg^−1^ min^−1^). This is an interesting finding and may also be of value for interventions in populations with restricted possibilities for exercise training, e.g. in hospital and rehabilitation settings. Conversely, athletes do not seem to profit from PHC. However, only four studies investigating athletes were included in the meta-analysis. Again considering the high risk of bias arising from lack of blinding here, one may hypothesise that non-athletes might be more prone to placebo effects from PHC compared with athletes who are likely aware of protocols with much longer exposure (i.e. LHTL) and hypoxic exercise training (i.e. LLTH).The meta-regression indicates that the design of the hypoxic intervention should be carefully planned since its features do independently predict the change in *V*O_2_max. The multivariate meta-regression indicates that longer and more frequent hypoxic exposure is more effective in increasing *V*O_2_max, probably by enhancing the hypoxic dose. PHC interventions involving more deoxygenation–reoxygenation cycles also appear to induce larger increases in *V*O_2_max, possibly illustrating the positive effect of this type of intermittent hypoxic exposure (e.g. improved mitochondrial function and antioxidative pathways [12]).

The risk of bias analysis revealed that very few studies applied blinding (Table 1), despite its vast importance. We acknowledge that the effort required to apply sham-hypoxia required to a control group is high, and even sometimes impossible (i.e. when investigating the effects of natural altitude). However, assessor blinding can be implemented relatively easily and would substantially increase the methodological quality and scientific rigor of the findings. Given the risk of confounding and bias in the included studies, there remains a need for well-designed, blinded and randomised trials investigating the effects of hypoxic training strategies on *V*O_2_max. In particular, studies should carefully manage and report the criteria used to measure *V*O_2_max according to the latest recommendations. The findings regarding risk of bias of our work are in line with a recent systematic review focusing on the methodological quality of studies investigating the acute effects of exercise under hypoxia, which rated overall methodology quality as fair [1]. In line with this work and earlier contributions [1, 19, 94], we emphasise on the importance of well-controlled, double-blind study designs in this field, addressing concerns regarding potential placebo effects.

This work has some limitations. First, we pooled the included studies according to the type of hypoxic exposure. A further stratification according to the types of training applied may be proposed (e.g. endurance versus strength training), for which we accounted by implementing a meta-regression. Second, as mentioned in the preceding paragraph, a majority of studies had methodological concerns. This may have led to biased estimates in our meta-analysis and meta-regression. Finally, it should be mentioned that studies mostly included males and young subjects (Table 1), and the effect of hypoxic interventions in females and older individuals requires further studies.

## Conclusions

Our meta-analysis showed significant effects on *V*O_2_max with LHTL in athletic populations and with LLTH and PHC in non-athletic populations. However, absolute differences in *V*O_2_max change are relatively small and suggest that hypoxic interventions only add slight benefits in comparison with control regarding the enhancement of maximal aerobic capacity. We identified the severity and exposure patterns of the intermittent hypoxic interventions as being associated with the amplitude of *V*O_2_max change following LLTH and PHC. Future studies should carefully consider and investigate these factors in order to establish the most appropriate interventions and candidates to benefit from hypoxic training. Since the risk of bias and confounding in the included studies was substantial, especially owing to the few studies applying blinding procedures, there remains a need for well-designed large-scale placebo-controlled trials focusing on the functional consequences of hypoxic training interventions.

## Supplementary Information

Below is the link to the electronic supplementary material.

Supplementary file1 (XLSX 681 KB)

Supplementary file2 (CSV 56 KB)

Supplementary file3 (R 23 KB)

Supplementary file4 (PDF 458 KB)

## Declarations

### Availability of data and material

All data supporting this work are available in the supplementary material.

### Code availability

Analysis codes are available in the supplementary material.
