# Supplementary material for: The Effects of Intermittent Hypoxic Training Strategies on Maximal Oxygen Uptake in Healthy Humans: A Meta-analysis with Meta-regression
Source: Sports Med. 2026 Sep 15;56(11):2767–82. doi: 10.1007/s40279-026-02482-9 (PMC13672411; doi:10.1007/s40279-026-02482-9)
Supplement: Supplementary file 1 [file 40279_2026_2482_MOESM4_ESM.pdf]

Supplementary Materials
The effects of intermittent hypoxic training strategies on maximal oxygen uptake in healthy humans: a meta-analysis with meta-regression

Running head: Hypoxic training strategies and VO_2max

Dario Kohlbrenner[1,2]*, Abdallah Ghaith[1]*, Joey Fournier[1], Charles Khouri[1], Antonin Bordas[1], Samarmar Chacaroun[1], Monique Mendelson[1], Patrice Flore[1], Mathieu Marillier[1], Julien Brugniaux[1], Jean-Louis Pépin[1], Renaud Tamisier[1], Samuel Verges[1]

*shared authorship based on equal contributions

[1]HP2 Laboratory, INSERM, Grenoble Alpes University, CHU Grenoble Alpes, Grenoble, France

[2]Epidemiology, Biostatistics, and Prevention Institute, University of Zurich, Zurich, Switzerland

Correspondence

Samuel Vergès, PhD

HP2 Laboratory, UM Sport Pathologies

Hôpital Sud, Avenue Kimberley

38434 Echirolles

France

sverges@chu-grenoble.fr

Search Strategy

Search strategy used for Pubmed:

(Hypoxia training[Tiab]) OR (hypoxic training[Tiab]) OR (intermittent hypoxia[Tiab]) OR (altitude training[Tiab]) OR (intermittent hypoxic[Tiab]) OR (hypoxic conditioning[Tiab]) OR (hypoxia conditioning[Tiab]) OR ("live high"[Tiab]) OR ("live low"[Tiab]) OR ("living high"[Tiab]) OR ("living low"[Tiab]) OR ("train high"[Tiab]) OR ("train low"[Tiab]) OR ("training high"[Tiab]) OR ("training low"[Tiab]) OR ("repetitive hypoxia"[Tiab]) OR ("repeated hypoxia"[Tiab]) OR ("cyclic hypoxia"[Tiab]) NOT (animal[Tiab]) NOT (animals[Tiab]) NOT (rodent[Tiab]) NOT (rodents[Tiab]) NOT (rat[Tiab]) NOT (rats[Tiab]) NOT (mice[Tiab]) NOT (mouse[Tiab]) NOT (fish[Tiab])

Search strategy used for Web of Science:

((TI=Hypoxia training OR AB=Hypoxia training) OR (TI=hypoxic training OR AB=hypoxic training) OR (TI=intermittent hypoxia OR AB=intermittent hypoxia) OR (TI=altitude training OR AB=altitude training) OR (TI=intermittent hypoxic OR AB=intermittent hypoxic) OR (TI=hypoxic conditioning OR AB=hypoxic conditioning) OR (TI=hypoxia conditioning OR AB=hypoxia conditioning) OR (TI="live high" OR AB="live high") OR (TI="live low" OR AB="live low") OR (TI="living high" OR AB="living high") OR (TI="train high" OR AB="train high") OR (TI="train low" OR AB="train low") OR (TI="training high" OR AB="training high") OR (TI="training low" OR AB="training low") OR (TI="living low" OR AB="living low") OR (TI="repetitive hypoxia" OR AB="repetitive hypoxia") OR (TI="repeated hypoxia" OR AB="repeated hypoxia") OR (TI="cyclic hypoxia" OR AB="cyclic hypoxia")) NOT (TI=animal OR AB=animal) NOT (TI=animals OR AB=animals) NOT (TI=rodent OR AB=rodent) NOT (TI=rodents OR AB=rodents) NOT (TI=rat OR AB=rat) NOT (TI=rats OR AB=rats) NOT (TI=mice OR AB=mice) NOT (TI=mouse OR AB=mouse) NOT (TI=fish OR AB=fish)

Search strategy used for Embase:

(Hypoxia AND training):ti,ab OR (hypoxic AND training):ti,ab OR (intermittent AND hypoxia):ti,ab OR (altitude AND training):ti,ab OR (intermittent AND hypoxic):ti,ab OR (hypoxic AND conditioning):ti,ab OR (hypoxia AND conditioning):ti,ab OR "live high":ti,ab OR "live low":ti,ab OR "living high":ti,ab OR "living low":ti,ab OR "train high":ti,ab OR "train low":ti,ab OR "training high":ti,ab OR "training low":ti,ab OR "repetitive hypoxia":ti,ab OR "repeated hypoxia":ti,ab OR "cyclic hypoxia":ti,ab NOT animal:ti,ab NOT animals:ti,ab NOT rodent:ti,ab NOT rodents:ti,ab NOT rat:ti,ab NOT rats:ti,ab NOT mice:ti,ab NOT mouse:ti,ab NOT fish:ti,ab

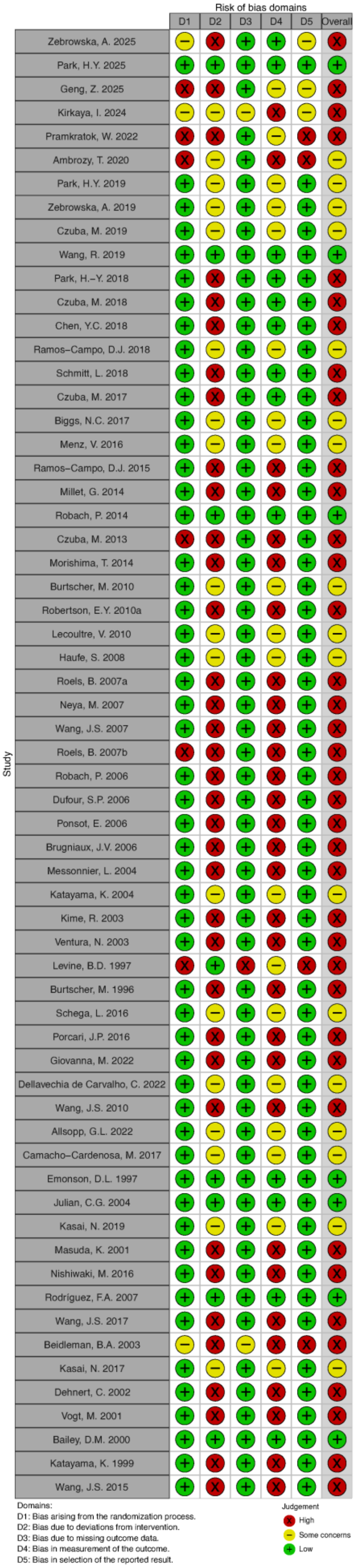
Risk of bias domains
D1
D2
D3
D4
D5
Overall
Study
Zebrowska, A. 2025
Park, H.Y. 2025
Geng, Z. 2025
Kirkaya, I. 2024
Pramkratok, W. 2022
Ambrozy, T. 2020
Park, H.Y. 2019
Zebrowska, A. 2019
Czuba, M. 2019
Wang, R. 2019
Park, H.–Y. 2018
Czuba, M. 2018
Chen, Y.C. 2018
Ramos–Campo, D.J. 2018
Schmitt, L. 2018
Czuba, M. 2017
Biggs, N.C. 2017
Menz, V. 2016
Ramos–Campo, D.J. 2015
Millet, G. 2014
Robach, P. 2014
Czuba, M. 2013
Morishima, T. 2014
Burtscher, M. 2010
Robertson, E.Y. 2010a
Lecoultre, V. 2010
Haufe, S. 2008
Roels, B. 2007a
Neya, M. 2007
Wang, J.S. 2007
Roels, B. 2007b
Robach, P. 2006
Dufour, S.P. 2006
Ponsot, E. 2006
Brugniaux, J.V. 2006
Messonnier, L. 2004
Katayama, K. 2004
Kime, R. 2003
Ventura, N. 2003
Levine, B.D. 1997
Burtscher, M. 1996
Schega, L. 2016
Porcari, J.P. 2016
Giovanna, M. 2022
Dellavechia de Carvalho, C. 2022
Wang, J.S. 2010
Allsopp, G.L. 2022
Camacho–Cardenosa, M. 2017
Emonson, D.L. 1997
Julian, C.G. 2004
Kasai, N. 2019
Masuda, K. 2001
Nishiwaki, M. 2016
Rodriguez, F.A. 2007
Wang, J.S. 2017
Beidleman, B.A. 2003
Kasai, N. 2017
Dehnert, C. 2002
Vogt, M. 2001
Bailey, D.M. 2000
Katayama, K. 1999
Wang, J.S. 2015
Domains:
D1: Bias arising from the randomization process.
D2: Bias due to deviations from intervention.
D3: Bias due to missing outcome data.
D4: Bias in measurement of the outcome.
D5: Bias in selection of the reported result.
Judgement
High
Some concerns
Low

Figure S1. Traffic light plot of the revised tool for assessing risk of bias in randomised trials (RoB2).

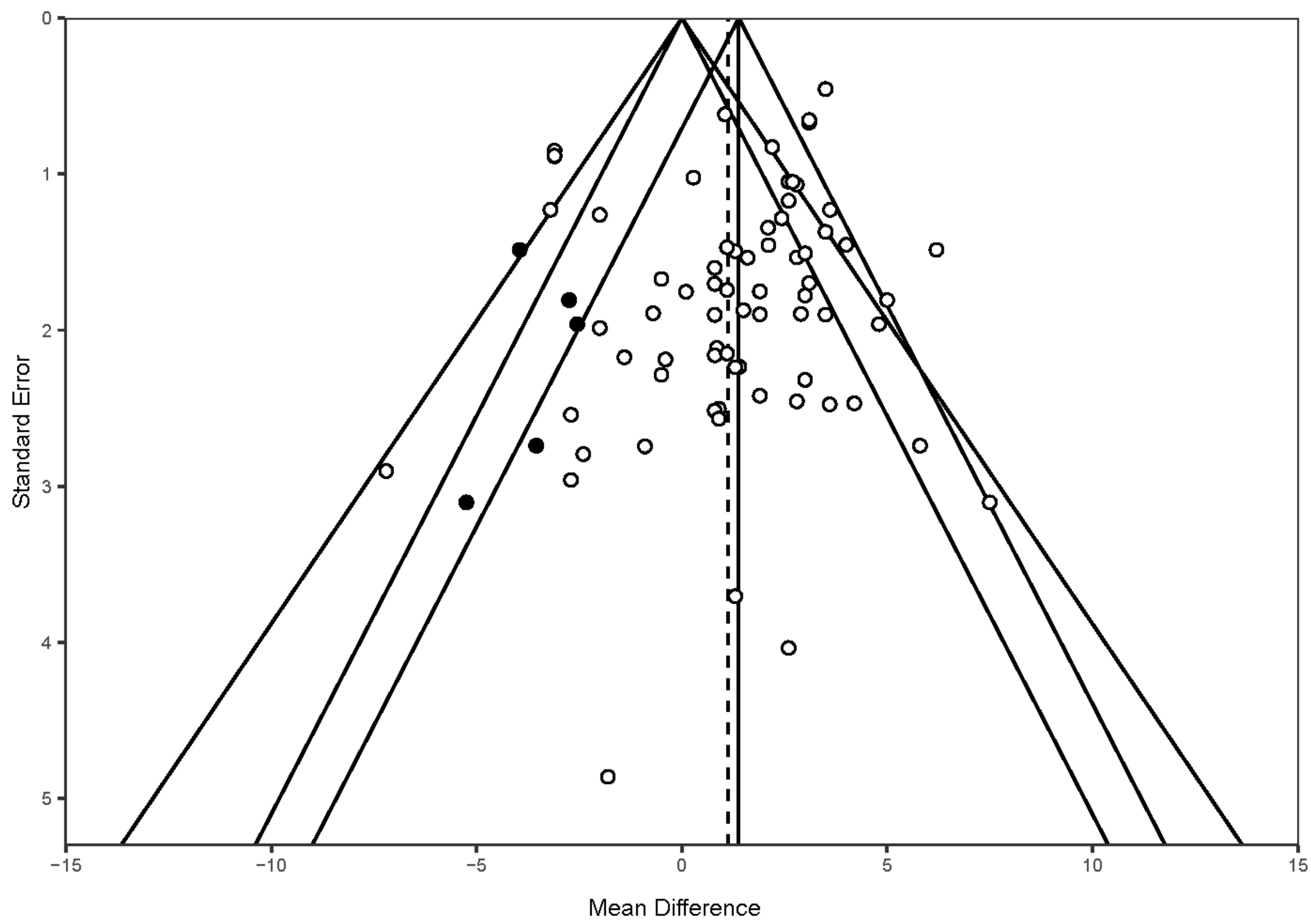

Figure S2. Funnel plot for the mean differences of the included studies. The standard error of each individual study is plotted against its mean difference.
